# Supplementary material for: Evidence for Non-optimal Codon Choice in Highly Transcribed Sex-Biased Genes of Drosophila melanogaster
Source: Genome Biol Evol. 2026 Apr 13;18(5):evag093. doi: 10.1093/gbe/evag093 (PMC13148536; doi:10.1093/gbe/evag093)
Supplement: evag093_Supplementary_Data [file evag093_supplementary_data.pdf]

SUPPLEMENTARY INFORMATION for

Evidence for non-optimal codon choice in highly transcribed sex-biased genes of *Drosophila melanogaster*

Carrie A. Whittle, Cassandra G. Extavour

Corresponding author: C.G. Extavour

Email: extavour@oeb.harvard.edu

Contents

Figs. S1 to S7

Tables S1 to S10

Supplementary text (Results and Discussion; Materials and Methods)

References for SI citations

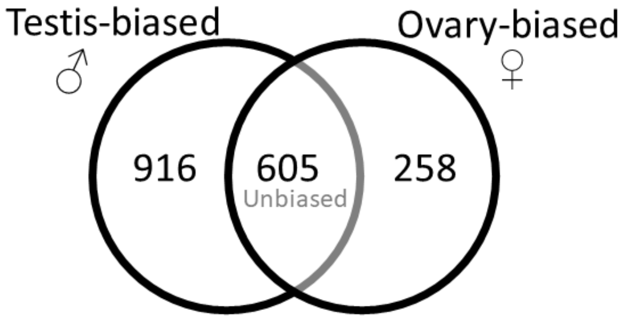

A. Number of sex-biased and unbiased genes

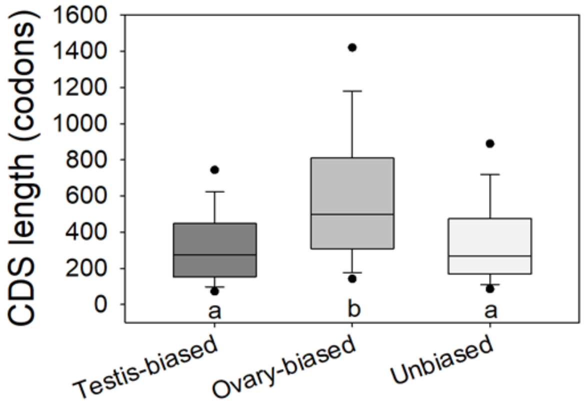

B. CDS length for sex-biased and unbiased genes

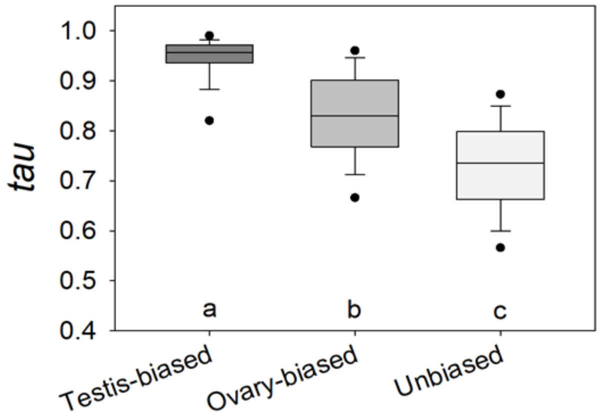

C. *tau* for sex-biased and unbiased genes

Fig. S1. (A) The number of sex-biased and unbiased genes under study. (B) CDS lengths per gene set. (C) the *tau* values per gene set. For A, the numbers of genes with testis-specific and ovary-specific expression (0 RPKM) with respect to the opposite sexual tissue was 724 (79.0%) and 19 (7.4%) respectively. Different letters under bars in B and C indicate a statistically significant difference with MWU-test $P<0.05$.

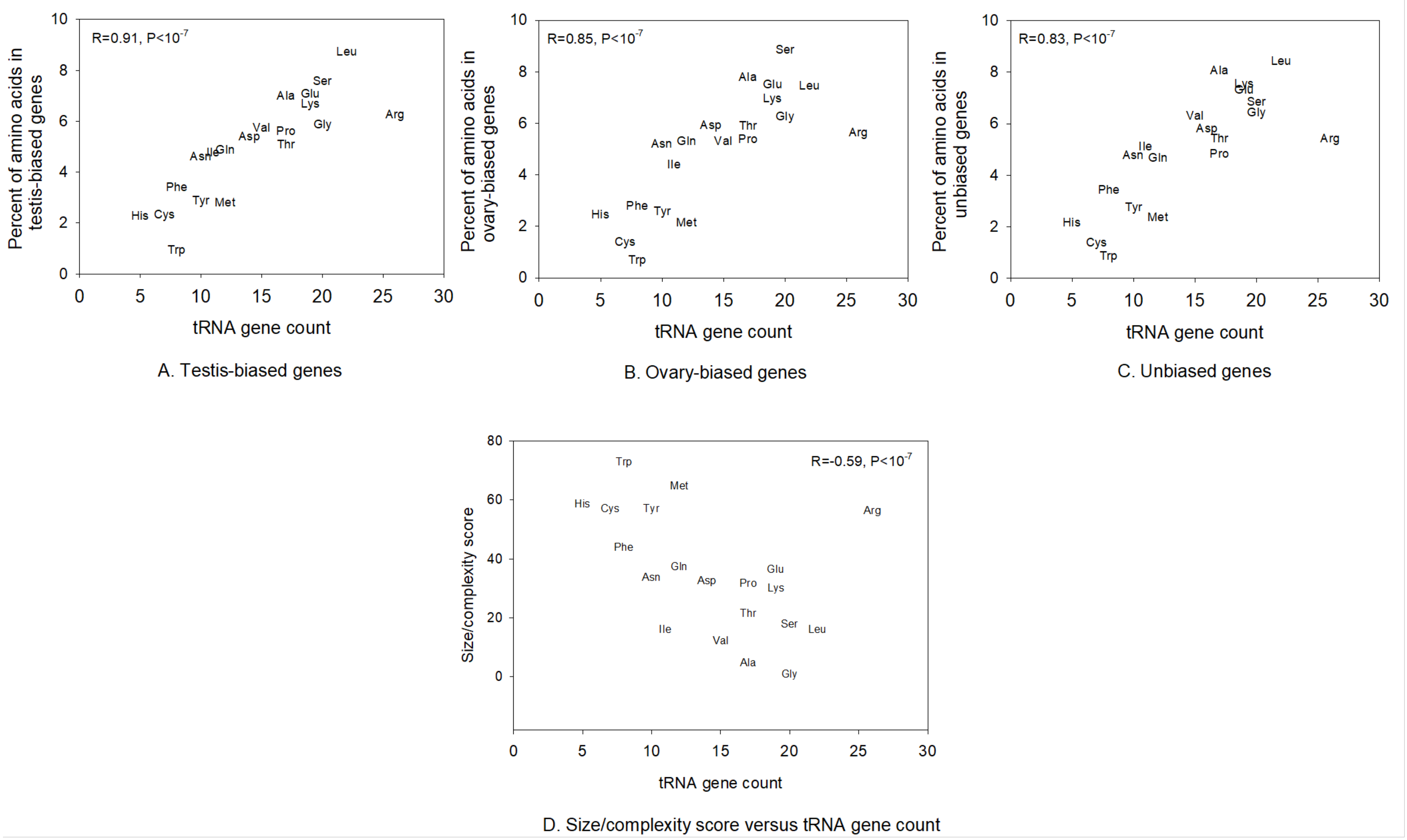

Fig. S2. The relationship between the frequency of amino acid use (percent of all concatenated amino acids per gene set) and tRNA genes in the genome of *D. melanogaster*. (A) testis-biased genes. (B) ovary-biased genes. (C) unbiased genes. (D) The association between the size/complexity score per amino acid and tRNA gene counts in the genome. The Spearman Rank correlations are shown. The number of amino acids for testis-biased genes was N=299,817, for ovary-biased genes N=155,282 amino acids, and for unbiased genes was N=218,580 amino acids.

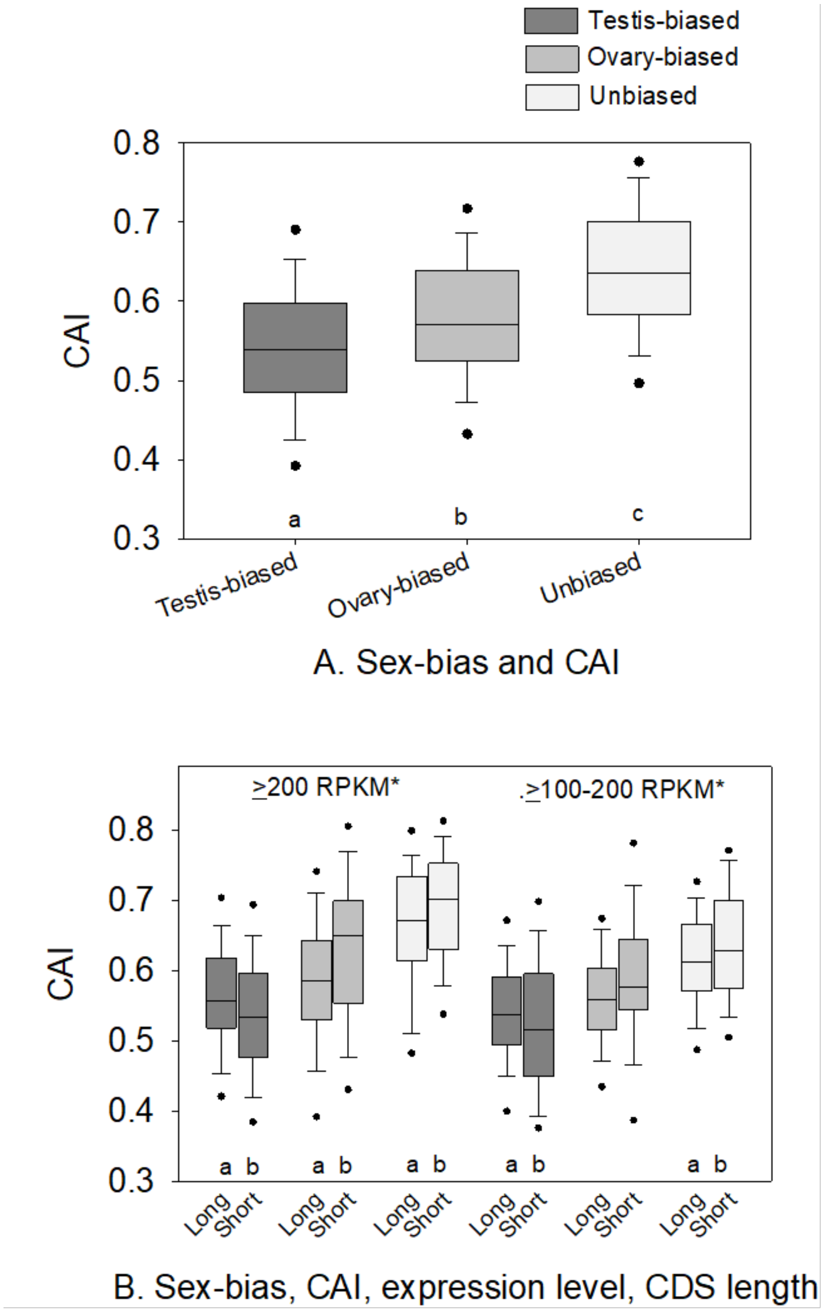

Fig. S3. Box plots of CAI for *D. melanogaster* sex-biased gene sets under study. (A) CAI of testis-biased, ovary-biased and unbiased genes. (B) CAI for testis-biased, ovary-biased and unbiased genes subdivided by gene expression level, and by CDS length. In A, different letters among bars indicates statistical significance using ranked ANOVA and Dunn's paired contrast (P<0.05). In panel B, different letters under each set of paired bars indicates CAI between short and long CDS had MWU-test P<0.05. *Indicates a difference in CAI between the high and low expressed genes for the testis-biased genes, the ovary-biased genes, and for the unbiased genes (MWU-test P<0.05; grouped genes across both length classes).

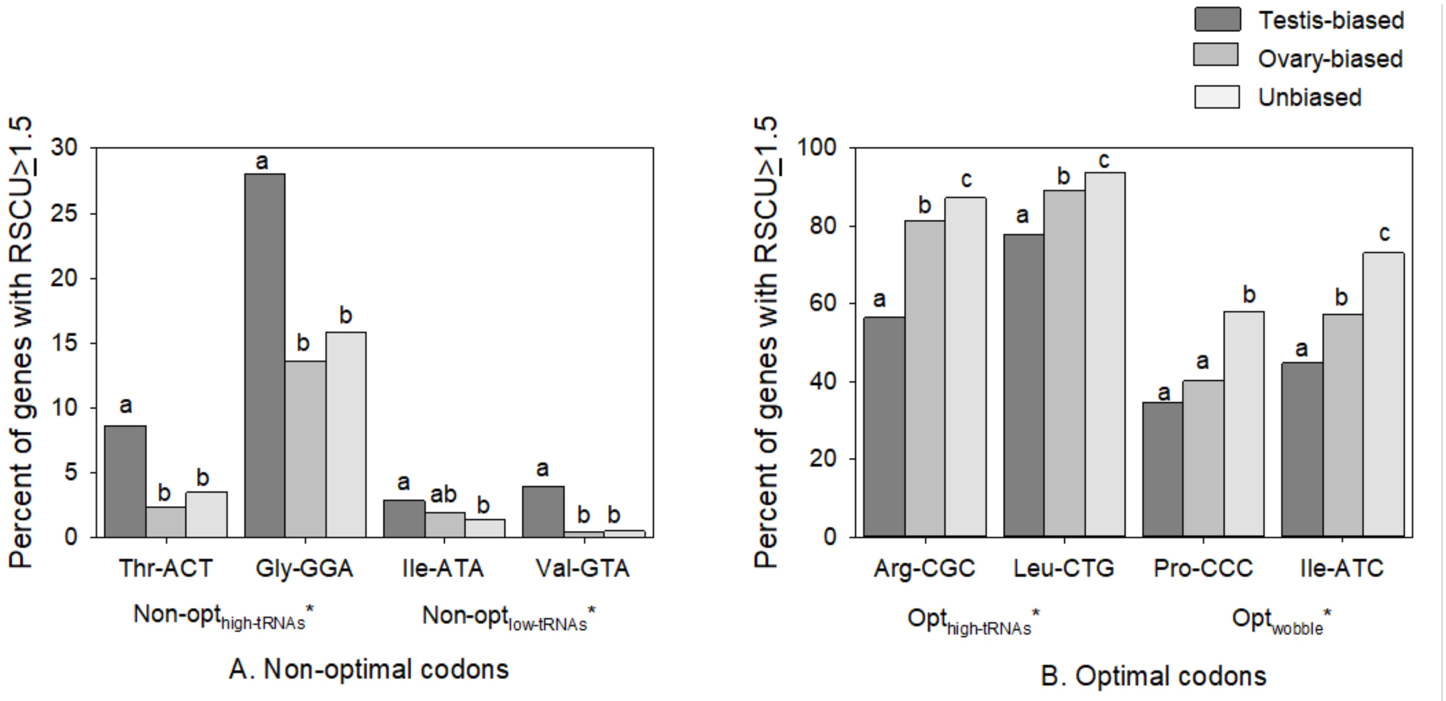

Fig. S4. Examples of non-optimal and optimal codons with very high use (RSCU≥1.5) in sex-biased and unbiased genes. (A) Four non-optimal codons, including two with high and two with a low number of matching tRNA genes. (B) optimal codons, including two with high numbers of matching tRNA genes and two requiring wobble tRNAs. In (A) and (B), different letters among the three bars for each codon indicates a statistically significant difference using Chi-square tests ($P<0.05$).* (asterisk) in (A) indicates percentages were higher for each Non-opt$_{\text{high-tRNAs}}$ codon versus each comparable Non-opt$_{\text{lowtRNAs}}$ codon for paired contrasts between testis-biased, ovary-biased and between unbiased genes (Chi-square $P<0.05$, with the only exception being between Thr-ACT and Ile-ATA for the ovary-biased genes $P>0.05$). * (asterisk) in (B) indicates all percentages were higher for each Opt$_{\text{high-tRNAs}}$ codon than each Opt$_{\text{wobble}}$ codon in paired contrasts between testis-biased genes, between ovary-biased genes and between unbiased genes (Chi-square $P<0.05$). The N for testis-biased genes =916, ovary-biased =258 and unbiased=605 genes. Note the Y-axis scales are different in (A) and (B).

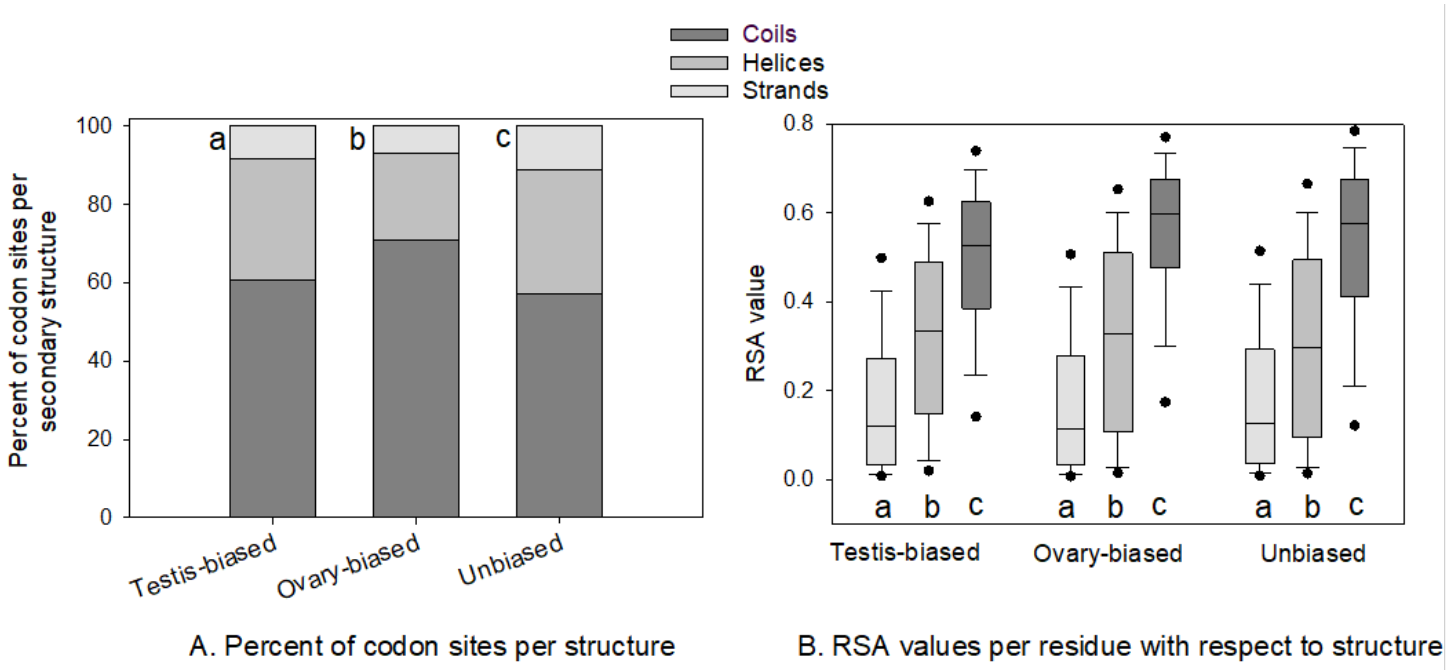

Fig. S5. (A) The percent of amino acids in sex-biased and unbiased genes located in sheets (strands), helices, and coils. (B) The RSA values for sex-biased and unbiased genes for all amino acids across all proteins. In (A), the percent in stands, helices and coils differed within all three gene sets (Chi-square tests P<0.05). In addition, a higher percemt of codons were located in coils for ovary-biased genes than testis-biased and unbiased genes (Chi-square test P<0.05). In (B), different letters within testis-biased, ovary-biased and unbiased genes indicates a statistically signficant difference in RSA using MWU-tests (P<0.05). All sites were concatenated across genes for each sex-biased gene category.

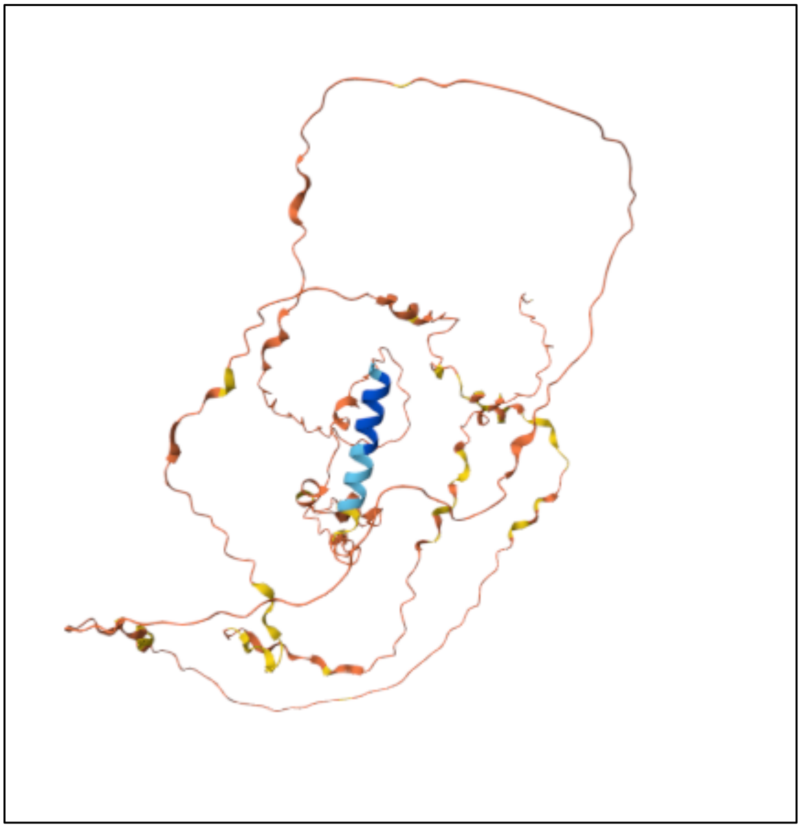

Fig. S6. An example of the predicted structure of a relatively long testis-bised gene protein (415 amino acids, protein ID=BG642163, Flybase ID= FBgn0083938) in the high Percent-Non-opt bin. Stucture is from Alpha-Fold (Jumper et al. 2021; Fleming et al. 2025) .

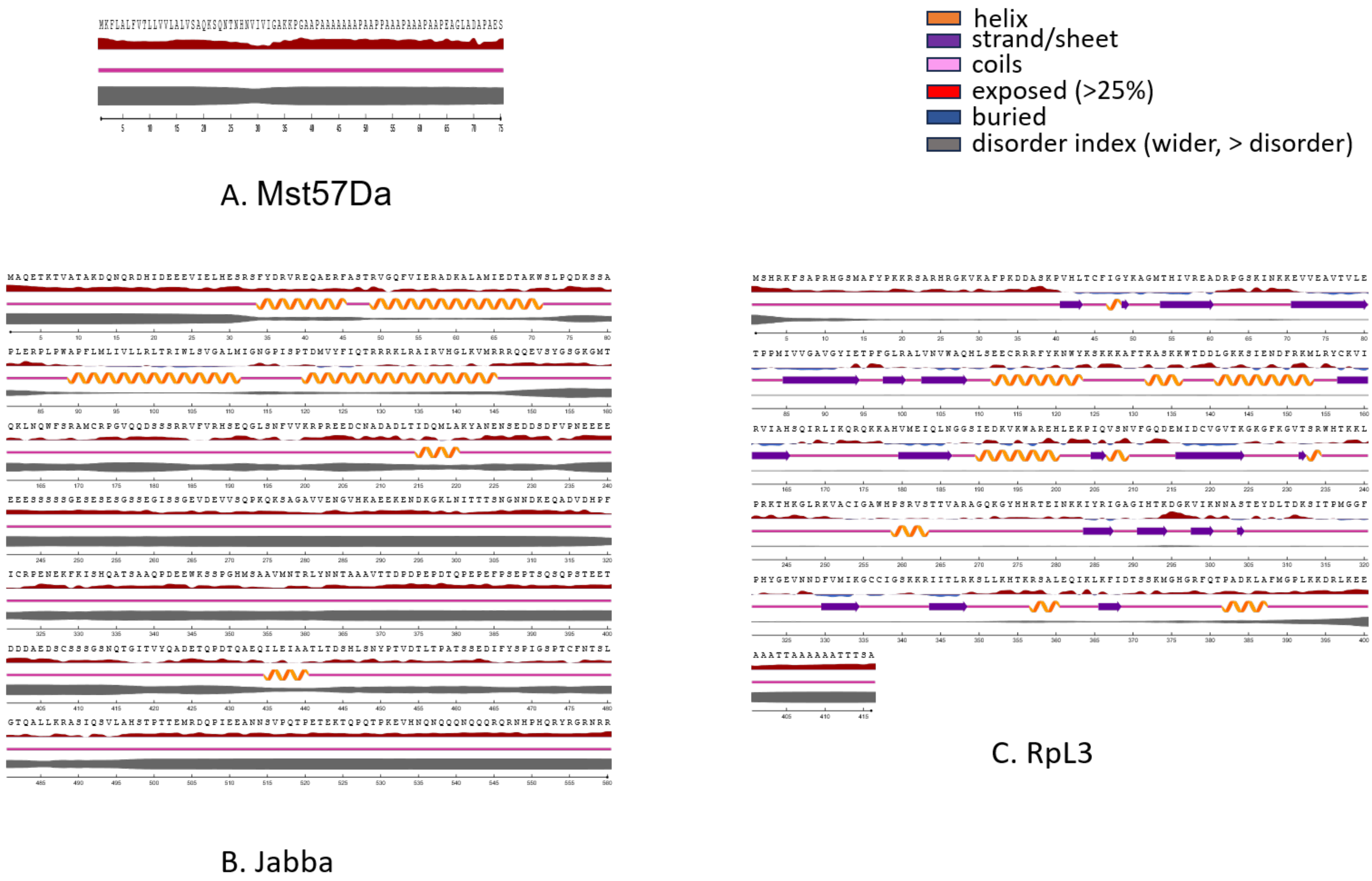

Fig. S7. Examples of the output of NetsurfP 3.0 (Hoie et al. 2022) for three genes provided in Fig. 3 G-I. RSA was determined for each amino acid based on its surface exposure.

Table S1. The organismal optimal and non-optimal codons for *D. melanogaster*. The ΔRSCU was determined between ribosomal genes versus all genes in the genome (ΔRSCU$_{Ribosome\text{-}All}$) and the number of exact matching tRNA genes were obtained from the GtRNA database (Chan and Lowe 2016). The codon statuses are defined in the main text and are as follows: Opt$_{high\text{-}tRNAs}$ = an optimal codon with abundant tRNA genes (≥5). Opt$_{wobble}$=an optimal codon requiring a wobble tRNA for translation, Non-opt$_{low\text{-}tRNAs}$= a non-optimal codon with few or no matching tRNA genes (<5), Non-opt$_{high\text{-}tRNAs}$= a non-optimal codon with a high number of matching tRNA genes (≥5). Optimal codons are in bold.

**Amino Acid**	**Codon**	**Dmel ribosomal RSCU**	**All genes RSCU**	**ΔRSCU Dmel**	**Optimal and non-optimal status**	**No. of tRNAs**	
						Anti-codon	No. tRNAs
Ala	GCT	0.85	0.79	+0.06	Non-opt$_{high\text{-}tRNAs}$	AGC	12
**Ala**	**GCC**	**2.14**	**1.75**	**+0.39**	**Opt$_{wobble}$**	**GGC**	**0**
Ala	GCA	0.44	0.71	-0.27	Non-opt$_{low\text{-}tRNAs}$	TGC	2
Ala	GCG	0.58	0.75	-0.17	Non-opt$_{low\text{-}tRNAs}$	CGC	3
Arg	CGT	1.42	0.93	+0.49	Non-opt$_{high\text{-}tRNAs}$	ACG	10
**Arg**	**CGC**	**2.67**	**1.86**	**+0.81**	**Opt$_{wobble}$**	**GCG**	**0**
Arg	CGA	0.48	0.95	-0.47	Non-opt$_{high\text{-}tRNAs}$	TCG	10
Arg	CGG	0.56	0.91	-0.35	Non-opt$_{low\text{-}tRNAs}$	CCG	0
Arg	AGA	0.31	0.61	-0.3	Non-opt$_{low\text{-}tRNAs}$	TCT	3
Arg	AGG	0.57	0.74	-0.17	Non-opt$_{low\text{-}tRNAs}$	CCT	3
Asn	AAT	0.63	0.93	-0.30	Non-opt$_{low\text{-}tRNAs}$	ATT	0
**Asn**	**AAC**	**1.37**	**1.07**	**+0.30**	**Opt$_{high\text{-}tRNAs}$**	GTT	**10**
Asp	GAT	0.94	1.08	-0.14	Non-opt$_{low\text{-}tRNAs}$	ATC	0
**Asp**	**GAC**	**1.06**	**0.92**	**+0.14**	**Opt$_{high\text{-}tRNAs}$**	**GTC**	**14**
Cys	TGT	0.39	0.61	-0.22	Non-opt$_{low\text{-}tRNAs}$	ACA	0
**Cys**	**TGC**	**1.61**	**1.39**	**+0.22**	**Opt$_{high\text{-}tRNAs}$**	GCA	**7**
Gln	CAA	0.47	0.63	-0.16	Non-opt$_{low\text{-}tRNAs}$	TTG	4
Gln	**CAG**	**1.53**	**1.37**	**+0.16**	**Opt$_{high\text{-}tRNAs}$**	CTG	**8**
Glu	GAA	0.54	0.7	-0.16	Non-opt$_{high\text{-}tRNAs}$	TTC	6
Glu	**GAG**	**1.46**	**1.3**	**+0.16**	**Opt$_{high\text{-}tRNAs}$**	**CTC**	**13**
Gly	GGT	0.99	0.86	+0.13	Non-opt$_{low\text{-}tRNAs}$	ACC	0

Gly	**GGC**	**1.81**	**1.66**	**+0.15**	**Opt$_{high\text{-}tRNAs}$**	**GCC**	**14**
Gly	GGA	1.02	1.18	-0.16	Non-opt$_{high\text{-}tRNAs}$	TCC	6
Gly	GGG	0.17	0.31	-0.14	Non-opt$_{low\text{-}tRNAs}$	CCC	0
His	CAT	0.65	0.81	-0.16	Non-opt$_{low\text{-}tRNAs}$	ATG	0
His	**CAC**	**1.35**	**1.19**	**+0.16**	**Opt$_{high\text{-}tRNAs}$**	**GTG**	**5**
Ile	ATT	0.92	1.04	-0.12	Non-opt$_{high\text{-}tRNAs}$	AAT	9
Ile	**ATC**	**1.77**	**1.35**	**+0.42**	**Opt$_{wobble}$**	**GAT**	**0**
Ile	ATA	0.31	0.62	-0.31	Non-opt$_{low\text{-}tRNAs}$	TAT	2
Leu	TTA	0.2	0.32	-0.12	Non-opt$_{low\text{-}tRNAs}$	TAA	4
Leu	TTG	0.91	1.1	-0.19	Non-opt$_{low\text{-}tRNAs}$	CAA	4
Leu	CTT	0.47	0.62	-0.15	Non-opt$_{low\text{-}tRNAs}$	AAG	4
Leu	CTC	0.9	0.91	-0.01	Non-opt$_{low\text{-}tRNAs}$	GAG	0
Leu	CTA	0.36	0.57	-0.21	Non-opt$_{low\text{-}tRNAs}$	TAG	2
Leu	**CTG**	**3.15**	**2.48**	**+0.67**	**Opt$_{high\text{-}tRNAs}$**	**CAG**	**8**
Lys	AAA	0.37	0.63	-0.26	Non-opt$_{high\text{-}tRNAs}$	TTT	6
**Lys**	**AAG**	**1.63**	**1.37**	**+0.26**	**Opt$_{high\text{-}tRNAs}$**	**CTT**	**13**
Met	ATG	1	1	0		CAT	12
Phe	TTT	0.52	0.79	-0.27	Non-opt$_{low\text{-}tRNAs}$	AAA	0
**Phe**	**TTC**	**1.48**	**1.21**	**+0.27**	**Opt$_{high\text{-}tRNAs}$**	**GAA**	**8**
Pro	CCT	0.48	0.54	-0.06	Non-opt$_{high\text{-}tRNAs}$	AGG	7
**Pro**	**CCC**	**1.82**	**1.29**	**+0.53**	**Opt$_{wobble}$**	**GGG**	**0**
Pro	CCA	0.7	1.03	-0.33	Non-opt$_{high\text{-}tRNAs}$	TGG	5
Pro	CCG	1	1.14	-0.14	Non-opt$_{high\text{-}tRNAs}$	CGG	5
Ser	TCT	0.53	0.53	0	Non-opt$_{high\text{-}tRNAs}$	AGA	8
Ser	**TCC**	**2.02**	**1.4**	**+0.62**	**Opt$_{wobble}$**	**GGA**	**0**
Ser	TCA	0.41	0.59	-0.18	Non-opt$_{low\text{-}tRNAs}$	TGA	2
Ser	TCG	1.17	1.16	0.01	Non-opt$_{low\text{-}tRNAs}$	CGA	4
Ser	AGT	0.5	0.87	-0.37	Non-opt$_{low\text{-}tRNAs}$	ACT	0
Ser	AGC	1.37	1.45	-0.08	Non-opt$_{high\text{-}tRNAs}$	GCT	6
Thr	ACT	0.57	0.71	-0.14	Non-opt$_{high\text{-}tRNAs}$	AGT	8
**Thr**	**ACC**	**2.03**	**1.47**	**+0.56**	**Opt$_{wobble}$**	**GGT**	**0**

Thr	ACA	0.52	0.82	-0.3	Non-opt$_{high\text{-}tRNAs}$	TGT	6
Thr	ACG	0.88	1	-0.12	Non-opt$_{low\text{-}tRNAs}$	CGT	3
Trp	TGG	1	1	0		CCA	8
Tyr	TAT	0.56	0.77	-0.21	Non-opt$_{low\text{-}tRNAs}$	ATA	0
**Tyr**	**TAC**	**1.44**	**1.23**	**+0.21**	**Opt$_{high\text{-}tRNAs}$**	**GTA**	**10**
Val	GTT	0.65	0.77	-0.12	Non-opt$_{high\text{-}tRNAs}$	AAC	6
Val	GTC	1.25	0.91	+0.34	Non-opt$_{low\text{-}tRNAs}$	GAC	0
Val	GTA	0.27	0.45	-0.18	Non-opt$_{low\text{-}tRNAs}$	TAC	2
**Val**	**GTG**	**1.84**	**1.86**	**-0.02**	**Opt$_{high\text{-}tRNAs}$**	**CAC**	**7**

Notes: A special case is Val, where GTG was classed as the optimal codon for analysis based on its much higher RSCU values than its sister codons (as observed for the other optimal codons) and which had high numbers of matching tRNA genes, while its ΔRSCU was near zero; thus GTC was classed as Non-opt$_{low\text{-}tRNAs}$, but this codon may also be interpreted as optimal (wobble) status. Assuming GTC to be the optimal codon had no consequence to our results and GTT and GTA remained non-optimal in both cases. All of the optimal codons shown were classed as optimal (preferred) at the CSD (Subramanian et al. 2022), had P-values<0.05 (that used the highest 10% ENC versus the lowest 10% ENC genes in the genome (Subramanian et al. 2022)), and also strongly agreed with the G3 and C3 optimal codons obtained from comparison of the highest and least expressed genes in *D. melanogaster* (Duret and Mouchiroud 1999). RSCU values represent the values across all concatenated genes for each gene set. Herein a single optimal codon was defined per amino acid using $\Delta RSCU_{Ribosome\text{-}All}$. Moreover, we classified all non-optimal codons (as all that were not the optimal codon, as described in Supplemental Text File S1), and every codon was also classified with respect to tRNA gene number status. N=169 for the ribosomal protein genes. The cutoff for classification as a high and low number of tRNA genes was ≥5 and <5 respectively (median of all tRNAs per gene was 4).

Table S2. The 30 developmental stages and 29 tissue types used for expression breadth analysis, or *tau*. The RPKM data is from Modencode (Li et al. 2014) and is available at FlyBase (Gramates et al. 2022). See also (Whittle and Extavour 2023). The 4d ovary and testis datasets used to assess sex-biased gene expression are included, and those used to assess 4-day virgin ovaries versus 4-day mated male accessory glands.

**Developmental stages**	**Tissue types**
em0-2hr	A_MateF_1d_head
em2-4hr	A_MateF_4d_ovary
em4-6hr	A_MateM_1d_head
em6-8hr	A_VirF_1d_head
em8-10hr	A_VirF_4d_head
em10-12hr	A_MateF_20d_head
em12-14hr	A_MateF_4d_head
em14-16hr	A_MateM_20d_head
em16-18hr	A_MateM_4d_acc_gland
em18-20hr	A_MateM_4d_head
em20-22hr	A_MateM_4d_testis
em22-24hr	A_1d_carcass
L1	A_1d_dig_sys
L2	A_20d_carcass
L3_12hr	A_20d_dig_sys
L3_PS1-2	A_4d_carcass
L3_PS3-6	A_4d_dig_sys
L3_PS7-9	P8_CNS
WPP	L3_CNS
P5	L3_Wand_carcass
P6	L3_Wand_dig_sys
P8	L3_Wand_fat
P9-10	L3_Wand_imag_disc
P15	L3_Wand_saliv
AdF_Ecl_1days	A_VirF_20d_head
AdF_Ecl_5days	A_VirF_4d_ovary
AdF_Ecl_30days	WPP_fat
AdM_Ecl_1days	WPP_saliv
AdM_Ecl_5days	P8_fat
AdM_Ecl_30days	

Table S3. Gene functional classification for all the testis-biased, ovary-biased and unbiased genes identified in *D. melanogaster*. Functions are from DAVID (Sherman et al. 2022). P-values indicate support of function from a Fisher's test. Genes could belong to more than one category.

Gene classification	Percent	P-Value
**Testis-biased**		
sexual reproduction	5.2	4.30E-20
multicellular organism reproduction	5.6	1.40E-18
Spermatogenesis	1.6	6.20E-11
sperm individualization	2	6.10E-10
tricarboxylic acid cycle	1.5	5.80E-09
sperm axoneme assembly	1.3	7.60E-09
mitochondrion	7.2	1.10E-08
cilium movement involved in cell motility	0.9	3.30E-08
carboxypeptidase activity	1	3.80E-08
sperm competition	1.1	6.40E-08
protein metabolic process	0.9	8.80E-08
motile cilium	1.3	1.80E-07
aminopeptidase activity	1	2.30E-07
protein import into mitochondrial matrix	1.2	3.50E-07
extracellular space	7	9.30E-07
**Ovary-biased**		
nucleus	45.3	7.90E-19
protein binding	25.2	8.80E-18
chorion	5.4	1.10E-12
mRNA binding	9.7	1.30E-11
nucleic acid binding	11.2	8.40E-09
RNA binding	13.2	1.20E-08
cytoplasm	32.9	2.90E-08
mitotic cell cycle	6.6	7.10E-08
vitelline envelope	2.3	9.10E-08
polytene chromosome	7	3.10E-07
nucleoplasm	8.9	5.60E-07
oogenesis	7.8	8.10E-07
vitelline membrane formation involved in chorion-containing eggshell formation	2.3	6.00E-06
chromosome condensation	3.5	1.90E-05
chromatin binding	5.8	2.20E-05
ribonucleoprotein complex	3.5	4.60E-05
**Unbiased**		
cytoplasmic translation	12.9	3.20E-84
cytosolic large ribosomal subunit	7.3	1.20E-46
cytosolic ribosome	11.3	2.80E-44
structural constituent of ribosome	13.2	1.50E-37

cytosolic small ribosomal subunit	5.7	1.50E-36
cytosol	28.2	6.30E-35
ribosome	11.5	2.90E-34
nuclear chromosome	5.7	2.50E-33
proteasome complex	4.5	3.00E-28
protein folding	5.6	1.80E-20
proteasome-mediated ubiquitin-dependent protein catabolic process	5.7	4.10E-19
cytoplasm	33	8.50E-19
DNA-templated transcription, initiation	3.7	4.70E-17
nucleoplasm	9.1	4.80E-17
proteasome regulatory particle	2.7	4.90E-17

Table S4. The size complexity (S/C) scores of amino acids (Dufton 1997).

Amino Acid	S/C score
Ala	4.76
Arg	56.34
Asn	33.72
Asp	32.72
Cys	57.16
Gln	37.48
Glu	36.48
Gly	1
His	58.7
Ile	16.04
Leu	16.04
Lys	30.14
Met	64.68
Phe	44
Pro	31.8
Ser	17.86
Thr	21.62
Trp	73
Tyr	57
Val	12.28

Table S5. The ΔRSCU$_{Testis\text{-}Ovary}$ and ΔRSCU$_{Testis\text{-}Unbiased}$ values in *D. melanogaster* for each of the 18 degenerate amino acids. The status of each codon per amino acid with respect to tRNA genes is shown. *P<0.05, **P<0.001. The primary, or most preferred, non-optimal codon per amino acid (in testis-biased genes) was defined as the codon with the largest positive ΔRSCU, which was typically the same codon identified in both ΔRSCU contrasts (in bold). In two cases, where the ranking of the largest positive codon varied between contrasts (Pro, Val) the codon with the largest average ΔRSCU was taken as the primary non-optimal codon. N/A=not applicable.

**Amino acid**	**Codon**	**ΔRSCU$_{Testis\text{-}Ovary}$**		**ΔRSCU$_{Testis\text{-}Unbiased}$**		**Codon Status**
		ΔRSCU	P	ΔRSCU	P	
Ala	GCT	+0.028		+0.061		Non-opt$_{high\text{-}tRNAs}$
Ala	GCC	-0.081	*	-0.392	**	Opt$_{wobble}$
Ala	**GCA**	**+0.021**		**+0.214**	**	**Non-opt$_{low\text{-}tRNAs}$**
Ala	GCG	+0.0015		+0.087	*	Non-opt$_{low\text{-}tRNAs}$
Arg	CGT	-0.110		-0.266	**	Non-opt$_{high\text{-}tRNAs}$
Arg	CGC	-0.577	**	-1.097	**	Opt$_{wobble}$
Arg	CGA	+0.076		+0.324	**	Non-opt$_{high\text{-}tRNAs}$
Arg	CGG	+0.067		+0.256	**	Non-opt$_{low\text{-}tRNAs}$
Arg	AGA	+0.187	**	+0.345	**	Non-opt$_{low\text{-}tRNAs}$
Arg	**AGG**	**+0.283**	**	**+0.370**	**	**Non-opt$_{low\text{-}tRNAs}$**
Asn	**AAT**	**+0.154**	**	**+0.355**	**	**Non-opt$_{low\text{-}tRNAs}$**
Asn	AAC	-0.175	**	-0.367	**	Opt$_{high\text{-}tRNAs}$
Asp	**GAT**	**+0.043**		**+0.191**	**	**Non-opt$_{low\text{-}tRNAs}$**
Asp	GAC	-0.071	**	-0.208	**	Opt$_{high\text{-}tRNAs}$
Cys	**TGT**	**+0.073**		**+0.226**	**	**Non-opt$_{low\text{-}tRNAs}$**
Cys	TGC	-0.104	**	-0.126	**	Opt$_{high\text{-}tRNAs}$
Gln	**CAA**	**+0.051**	*	**+0.201**	**	**Non-opt$_{low\text{-}tRNAs}$**
Gln	CAG	-0.087	**	-0.240	**	Opt$_{high\text{-}tRNAs}$
Glu	**GAA**	**+0.096**	**	**+0.232**	**	**Non-opt$_{high\text{-}tRNAs}$**
Glu	GAG	-0.139	**	-0.262	**	Opt$_{high\text{-}tRNAs}$
Gly	GGT	-0.034		+0.013		Non-opt$_{low\text{-}tRNAs}$
Gly	GGC	-0.289	**	-0.450	**	Opt$_{high\text{-}tRNAs}$
Gly	**GGA**	**+0.204**	**	**+0.276**	**	**Non-opt$_{high\text{-}tRNAs}$**
Gly	GGG	+0.115	*	+0.170	**	Non-opt$_{low\text{-}tRNAs}$

His	**CAT**	**+0.064**		**+0.159**	**	**Non-opt$_{\text{low-tRNAs}}$**
His	CAC	-0.142	**	-0.235	**	Opt$_{\text{high-tRNAs}}$
Ile	ATT	+0.012		+0.109	**	Non-opt$_{\text{high-tRNAs}}$
Ile	ATC	-0.198	**	-0.437	**	Opt$_{\text{wobble}}$
Ile	**ATA**	**+0.114**		**+0.266**	**	**Non-opt$_{\text{low-tRNAs}}$**
Leu	TTA	+0.036		+0.154	**	Non-opt$_{\text{low-tRNAs}}$
Leu	**TTG**	**+0.142**	*	**+0.259**	**	**Non-opt$_{\text{low-tRNAs}}$**
Leu	CTT	+0.066		+0.189	**	Non-opt$_{\text{low-tRNAs}}$
Leu	CTC	-0.002		-0.046		Non-opt$_{\text{low-tRNAs}}$
Leu	CTA	+0.046		+0.197	**	Non-opt$_{\text{low-tRNAs}}$
Leu	CTG	-0.353	**	-0.818	**	Opt$_{\text{high-tRNAs}}$
Lys	**AAA**	**+0.026**		**+0.161**	**	**Non-opt$_{\text{high-tRNAs}}$**
Lys	AAG	-0.070	**	-0.213	**	Opt$_{\text{high-tRNAs}}$
Met	ATG	-	-	N/A		
Phe	**TTT**	**+0.086**		**+0.291**	**	**Non-opt$_{\text{low-tRNAs}}$**
Phe	TTC	-0.120	**	-0.296	**	Opt$_{\text{high-tRNAs}}$
Pro	**CCT**	**+0.090**	*	**+0.149**	*	**Non-opt$_{\text{high-tRNAs}}$**
Pro	CCC	-0.150	*	-0.528	**	Opt$_{\text{wobble}}$
Pro	CCA	0		+0.235	**	Non-opt$_{\text{high-tRNAs}}$
Pro	CCG	0	*	+0.135	*	Non-opt$_{\text{high-tRNAs}}$
Ser	TCT	+0.047	*	+0.091	*	Non-opt$_{\text{high-tRNAs}}$
Ser	TCC	+0.039		-0.399	**	Opt$_{\text{wobble}}$
Ser	TCA	+0.021		+0.176	**	Non-opt$_{\text{low-tRNAs}}$
Ser	TCG	-0.150	*	-0.231	**	Non-opt$_{\text{low-tRNAs}}$
Ser	**AGT**	**+0.214**	**	**+0.402**	**	**Non-opt$_{\text{low-tRNAs}}$**
Ser	AGC	-0.196	*	-0.064		Non-opt$_{\text{high-tRNAs}}$
Thr	**ACT**	**+0.166**	**	**+0.201**	**	**Non-opt$_{\text{high-tRNAs}}$**
Thr	ACC	-0.037		-0.334	**	Opt$_{\text{wobble}}$
Thr	ACA	-0.111	*	+0.082	*	Non-opt$_{\text{high-tRNAs}}$
Thr	ACG	-0.051	*	+0.042		Non-opt$_{\text{low-tRNAs}}$

Trp	TGG	-	-	N/A		
Tyr	**TAT**	**+0.094**		**+0.284**	**	**Non-opt$_{\mathbf{low\text{-}tRNAs}}$**
Tyr	TAC	-0.174	**	-0.308	**	Opt$_{high\text{-}tRNAs}$
Val	GTT	+0.042		+0.157	**	Non-opt$_{high\text{-}tRNAs}$
Val	GTC	-0.061		-0.140	*	Non-opt$_{low\text{-}tRNAs}$
Val	**GTA**	**+0.066**		**+0.148**	**	**Non-opt$_{\mathbf{low\text{-}tRNAs}}$**
Val	GTG	-0.089	**	-0.208	**	Opt$_{high\text{-}tRNAs}$

Table S6. Gene functional clustering for the 212 testis-biased genes with high use of non-optimal codons in *D. melanogaster* from Fig. 2. Functions are from DAVID (Sherman et al. 2022). P-values are derived from a modified Fisher's test, where lower values indicate greater enrichment.

**Enrichment Score: 13.43**	**No. genes**	**P-Value**
sexual reproduction	25	3.50E-23
multicellular organism reproduction	25	4.40E-21
extracellular space	27	4.50E-11
**Enrichment Score: 3.02**		
behavior	6	5.50E-06
secreted	9	2.90E-03
extracellular region	10	5.50E-02
**Enrichment Score: 2.49**		
spermatogenesis	15	4.40E-06
sperm axoneme assembly	4	7.60E-04
differentiation	6	5.30E-03
**Enrichment Score: 2.2**		
microtubule plus-end	4	3.90E-04
microtubule-associated protein RP/EB	3	6.30E-04
protein localization to microtubule	3	6.60E-04
regulation of microtubule polymerization or depolymerization	3	1.60E-03
microtubule plus-end binding	3	2.00E-03
spindle midzone	3	1.60E-02
cytoplasmic microtubule	3	1.60E-02
calponin homology domain	3	1.90E-02
spindle assembly	3	2.80E-02
microtubule organizing center	3	6.30E-02
microtubule binding	3	9.70E-02

Table S7. Examples of testis-biased genes with very high non-optimal codon use (>60%). Note Met and Trp were excluded in percentage calculations (see Supplementary Text File 1).

FlyBase ID	Gene name	Percent non-optimal codons
FBgn0083938	*BG642163*	82.21
FBgn0083936	*Acp54A1*	76.19
FBgn0250831	*BG642167*	74.75
FBgn0004414	*msopa*	74.39
FBgn0011668	*Mst57Da*	72.97
FBgn0028412	*Mst33A*	72.41
FBgn0261055	*Sfp26Ad*	72.03
FBgn0002855	*Acp26Aa*	70.77
FBgn0011559	*Acp36DE*	70.59
FBgn0011239	*ms(2)35Ci*	70.40
FBgn0047334	*BG642312*	70.27
FBgn0034152	*Acp53C14a*	69.75
FBgn0051281	*Tpl94D*	68.15
FBgn0001281	*janB*	67.88
FBgn0015584	*Acp53Ea*	67.52
FBgn0004175	*Mst84Dd*	67.14
FBgn0032269	*w-cup*	66.33
FBgn0259975	*Sfp87B*	66.28
FBgn0004174	*Mst84Dc*	66.04
FBgn0004173	*Mst84Db*	65.75
FBgn0050365	*spaw*	65.49
FBgn0036970	*Spn77Bc*	65.42
FBgn0250832	*Dup99B*	64.71
FBgn0020399	*Mst89B*	64.07
FBgn0011670	*Mst57Dc*	63.89
FBgn0000592	*Est-6*	63.71
FBgn0013300	*Mst35Ba*	63.57
FBgn0039124	*tbrd-1*	62.60
FBgn0038281	*RpL10Aa*	62.38
FBgn0067903	*IM18*	62.32
FBgn0003742	*tra2*	62.07
FBgn0031367	*c-cup*	61.34
FBgn0043025	*Msi*	61.28
FBgn0032660	*elfless*	61.16
FBgn0046874	*Pif1B*	60.93
FBgn0019828	*dj*	60.83
FBgn0001099	*gdl*	60.64
FBgn0031878	*sip2*	60.48
FBgn0038225	*soti*	60.14

Table S8. The average RSA of amino acids from testis-biased genes when encoded by the primary non-optimal codon (defined in Table S5) versus the optimal codon (Table S1) for all 18 degenerate amino acids. Standard errors (SE) are shown. Sign test P<0.001.

	Primary non-optimal codon				Optimal codon				
**Amino acid**	**Codon**	**Average RSA**	**SE**	**Codon status**	**Codon**	**Average RSA**	**SE**	**Codon status**	**Sign test (+/-)**
Ala	GCA	0.397	0.003	Non-optlow-tRNAs	GCC	0.330	0.002	Optwobble	+
Arg	AGG	0.496	0.003	Non-optlow-tRNAs	CGC	0.472	0.002	Optwobble	+
Asn	AAT	0.540	0.002	Non-optlow-tRNAs	AAC	0.521	0.002	Opthigh-tRNAs	+
Asp	GAT	0.538	0.002	Non-optlow-tRNAs	GAC	0.529	0.002	Opthigh-tRNAs	+
Cys	TGT	0.244	0.003	Non-optlow-tRNAs	TGC	0.226	0.002	Opthigh-tRNAs	+
Gln	CAA	0.539	0.002	Non-optlow-tRNAs	CAG	0.516	0.002	Opthigh-tRNAs	+
Glu	GAA	0.577	0.002	Non-opthigh-tRNAs	GAG	0.555	0.001	Opthigh-tRNAs	+
Gly	GGA	0.448	0.003	Non-opthigh-tRNAs	GGC	0.426	0.003	Opthigh-tRNAs	+
His	CAT	0.441	0.003	Non-optlow-tRNAs	CAC	0.427	0.003	Opthigh-tRNAs	+
Ile	ATA	0.253	0.003	Non-optlow-tRNAs	ATC	0.219	0.002	Optwobble	+
Leu	TTG	0.273	0.003	Non-optlow-tRNAs	CTG	0.244	0.002	Opthigh-tRNAs	+
Lys	AAA	0.564	0.002	Non-opthigh-tRNAs	AAG	0.547	0.001	Opthigh-tRNAs	+
Phe	TTT	0.268	0.003	Non-optlow-tRNAs	TTC	0.239	0.002	Opthigh-tRNAs	+
Pro	CCT	0.498	0.003	Non-opthigh-tRNAs	CCC	0.455	0.002	Optwobble	+
Ser	AGT	0.488	0.003	Non-optlow-tRNAs	TCC	0.452	0.003	Optwobble	+
Thr	ACT	0.447	0.004	Non-opthigh-tRNAs	ACC	0.411	0.002	Optwobble	+
Tyr	TAT	0.308	0.003	Non-optlow-tRNAs	TAC	0.292	0.002	Opthigh-tRNAs	+
Val	GTA	0.303	0.004	Non-optlow-tRNAs	GTG	0.263	0.002	Opthigh-tRNAs	+
									18 of 18 amino acids positive<br>P<0.001

Table S9. RSCU values used to determine ΔRSCU for testis-biased versus ovary-biased genes.

Codon	Amino acid	RSCU testis (average)	RSCU ovary (average)
GCT	Ala	0.770229	0.742442
GCC	Ala	1.793483	1.875
GCA	Ala	0.696605	0.675581
GCG	Ala	0.708843	0.707248
CGT	Arg	0.964476	1.074574
CGC	Arg	1.746725	2.32469
CGA	Arg	0.85952	0.783915
CGG	Arg	0.866714	0.799419
AGA	Arg	0.637085	0.449922
AGG	Arg	0.82798	0.545155
AAT	Asn	0.9275	0.773721
AAC	Asn	1.050873	1.226395
GAT	Asp	1.040055	0.996628
GAC	Asp	0.916441	0.987984
TGT	Cys	0.560273	0.487403
TGC	Cys	1.291321	1.396318
CAA	Gln	0.614028	0.562946
CAG	Gln	1.333723	1.421628
GAA	Glu	0.692697	0.596705
GAG	Glu	1.263821	1.403411
GGT	Gly	0.838723	0.873643
GGC	Gly	1.549574	1.838605
GGA	Gly	1.222249	1.018333
GGG	Gly	0.385011	0.269574
CAT	His	0.758493	0.694884
CAC	His	1.108384	1.250969
ATT	Ile	0.975993	0.963527
ATC	Ile	1.373308	1.572054
ATA	Ile	0.579017	0.465155
TTA	Leu	0.30036	0.26438
TTG	Leu	1.156441	1.014341
CTT	Leu	0.646277	0.580155
CTC	Leu	0.940644	0.942752
CTA	Leu	0.549214	0.50314
CTG	Leu	2.342544	2.696124
AAA	Lys	0.566736	0.54062
AAG	Lys	1.381015	1.451783
ATG	Met	N/A	N/A
TTT	Phe	0.751616	0.665814
TTC	Phe	1.21357	1.334419
CCT	Pro	0.542686	0.452481
CCC	Pro	1.30238	1.452674
CCA	Pro	0.973624	0.974225

CCG	Pro	1.119574	1.119845
TCT	Ser	0.560437	0.513798
TCC	Ser	1.46524	1.426395
TCA	Ser	0.558854	0.537752
TCG	Ser	1.171463	1.321783
AGT	Ser	0.858439	0.644302
AGC	Ser	1.360033	1.556357
ACT	Thr	0.792587	0.62686
ACC	Thr	1.624902	1.662752
ACA	Thr	0.647041	0.758605
ACG	Thr	0.900022	0.952016
TGG	Trp	N/A	N/A
TAT	Tyr	0.741681	0.647364
TAC	Tyr	1.171103	1.345194
GTT	Val	0.767544	0.725271
GTC	Val	0.970731	1.032171
GTA	Val	0.455197	0.389109
GTG	Val	1.749323	1.838605

NA=not applicable. Note that RSCU values per codon
for each amino acid are averages across all genes under study.

Table S10. RSCU values used to determine ΔRSCU for testis-biased versus unbiased genes.

Codon	Amino acid	RSCU-testis (average)	RSCU unbiased (average)
GCT	Ala	0.770229	0.709157
GCC	Ala	1.793483	2.185702
GCA	Ala	0.696605	0.482926
GCG	Ala	0.708843	0.622281
CGT	Arg	0.964476	1.231091
CGC	Arg	1.746725	2.84443
CGA	Arg	0.85952	0.535769
CGG	Arg	0.866714	0.610777
AGA	Arg	0.637085	0.291901
AGG	Arg	0.82798	0.457207
AAT	Asn	0.9275	0.572033
AAC	Asn	1.050873	1.418182
GAT	Asp	1.040055	0.848727
GAC	Asp	0.916441	1.124975
TGT	Cys	0.560273	0.334116
TGC	Cys	1.291321	1.41795
CAA	Gln	0.614028	0.412545
CAG	Gln	1.333723	1.574413
GAA	Glu	0.692697	0.461157
GAG	Glu	1.263821	1.525835
GGT	Gly	0.838723	0.825983
GGC	Gly	1.549574	2
GGA	Gly	1.222249	0.946116
GGG	Gly	0.385011	0.214992
CAT	His	0.758493	0.599537
CAC	His	1.108384	1.344314
ATT	Ile	0.975993	0.866727
ATC	Ile	1.373308	1.810678
ATA	Ile	0.579017	0.313322
TTA	Leu	0.30036	0.145901
TTG	Leu	1.156441	0.897388
CTT	Leu	0.646277	0.456975
CTC	Leu	0.940644	0.986727
CTA	Leu	0.549214	0.353554
CTG	Leu	2.342544	3.160926
AAA	Lys	0.566736	0.405851
AAG	Lys	1.381015	1.594298
ATG	Met	N/A	N/A
TTT	Phe	0.751616	0.460413
TTC	Phe	1.21357	1.510083
CCT	Pro	0.542686	0.393421
CCC	Pro	1.30238	1.830612
CCA	Pro	0.973624	0.738529

CCG	Pro	1.119574	0.984083
TCT	Ser	0.560437	0.46914
TCC	Ser	1.46524	1.864364
TCA	Ser	0.558854	0.383107
TCG	Ser	1.171463	1.402694
AGT	Ser	0.858439	0.456777
AGC	Ser	1.360033	1.424744
ACT	Thr	0.792587	0.591471
ACC	Thr	1.624902	1.95914
ACA	Thr	0.647041	0.564942
ACG	Thr	0.900022	0.857669
TGG	Trp	N/A	N/A
TAT	Tyr	0.741681	0.457471
TAC	Tyr	1.171103	1.479818
GTT	Val	0.767544	0.610198
GTC	Val	0.970731	1.111058
GTA	Val	0.455197	0.307157
GTG	Val	1.749323	1.958231

NA=not applicable. Note that RSCU values per codon for each amino acid are averages across =all genes under study.

Supplementary Text File S1

Supplementary Results and Discussion

Here, we provide additional details on topics discussed in the main text Results and Discussion.

Identification and Characterization of the Sex-Biased Genes: Distribution of Sex-Biased Genes Across Chromosomes

The sex-biased and unbiased genes were largely similar in distribution among autosomes 2 and 3 in *D. melanogaster* (respectively, 45.0 and 46.5% of testis-biased genes, 38.7 and 45.6% of unbiased genes, and 34.5 and 36.4% of ovary-biased; less than 1% on the micro-chromosome 4). However an elevated percentage of the ovary-biased gene set was located on the X-chromosome (N=72 genes, or 27.9%) as compared to the testis-biased (N=75 or 8.2%) or unbiased genes (N=91, or 15.0%), possibly reflecting weak dosage compensation on the X chromosome in male gonads (and thus low propensity for male-bias), or innate benefits of the location of female-biased genes on the X chromosome as they are carried in females more often than autosomes (Parisi et al. 2003; Argyridou and Parsch 2018; Whittle et al. 2020).

Optimal and Non-optimal Codon Identified in Other *Drosophila melanogaster* subgroup species

It is worth noting that while we focused on *D. melanogaster* herein due to the extent of publicly available genomic and transcriptome datasets and gene characterization for this species, we found that four of its sister *melanogaster* subgroup species (*D. simulans, D. sechellia*, *D. yakuba* and *D. erecta*) had the exact same identity of optimal codons and non-optimal codons (Table S1), and the RSCU values and tRNA gene counts used to identify optimal/non-optimal statuses were strongly correlated to *D. melanogaster* (Spearman's R of the RSCU values within highly expressed ribosomal protein genes across all codons was $R\geq0.990$ ($P<10^{-7}$ and for tRNA gene counts per codon was $R\geq0.998$ ($P<10^{-7}$) suggesting the patterns we report herein for *D. melanogaster*, may also apply to its four related sister species.

Identification and Characterization of the Sex-Biased Genes: CAI and expression level and CDS length (Fig. S3)

We found a stepwise decrease in CAI from unbiased, to ovary-biased to testis-biased genes (Ranked ANOVA and Dunn's P<0.05, Fig. S3A), thus corresponding to a marked increase in non-optimal codons in the testis-biased genes. The pattern in CAI differs from prior suggestions, based on analysis solely of optimal codon use (without tRNA analysis, or non-optimal analysis in those studies), that ovary-biased and unbiased genes in *D. melanogaster* had similar Fop values (Zhang et al. 2004; Hambuch and Parsch 2005). We suggest that our results likely have greater sensitivity than previous studies, as we obtained our CAI values by using genome-wide RNA-seq herein (Graveley et al. 2011; Li et al. 2014; Gramates et al. 2022), rather than partial microarrays as done for previous studies (Zhang et al. 2004; Hambuch and Parsch 2005). Furthermore, the study of

only unbiased genes that were substantially expressed within the sexual tissue types (≥100 RPKM in at least one sexual tissue (and less than 5 fold difference between sexes), see Materials and Methods) exposes a heightened tendency for optimal codon use in unbiased genes. Importantly, Fig. S3A demonstrates a marked elevation in non-optimal codon use in the testis-biased genes, as compared to ovary-biased and unbiased genes.

Given that expression level and CDS length have each been associated with optimal codon use in various organisms (Comeron et al. 1999; Duret and Mouchiroud 1999; Akashi 2001; Hambuch and Parsch 2005; Ingvarsson 2007; Whittle et al. 2007; Whittle et al. 2019; Whittle et al. 2021), and thus, indirectly linked to non-optimal codon use, we subdivided the gene sets by expression level (two classes, ≥200 RPKM, and ≥100-200 RPKM; note that all genes under study had ≥100RPKM in the tissue with sex-biased expression and thus none were classed as having low expression; the average testis and ovary RPKM was used for this assessment for unbiased genes) and into short and long CDS lengths, with long defined as ≥272 codons, which represents the lowest 33rd percentile of the genome-wide CDS lengths. We found that the ≥200 RPKM expressed genes had an elevated CAI as compared to the ≥100-200 RPKM group for testis-biased, ovary-based and for unbiased genes (MWU-tests $P<0.05$, Fig. S3B). With respect to CDS length, short CDS genes had higher CAI than long CDS, for ovary-biased and unbiased genes (MWU-tests $P<0.05$, Fig. S3B). However, an opposite pattern was found for testis-biased genes, where the short CDS genes had markedly lower CAI than the long CDS, for both expression classes (MWU-tests $P<0.05$ for each contrast), demonstrating a unique pattern of ameliorated non-optimal codon use in short testis-biased CDS. While it may be suggested that that lower optimal codons in testis-biased genes (Fig. S3A) may be due to genetic inference (background selection or selective sweeps (Hill and Robertson 1966)) from linked adaptive amino acid substitutions causing fixation of linked non-optimal codons (Betancourt and Presgraves 2002), interference may be expected to reduce optimal codon use (and elevate non-optimal use) in longer CDS, rather than shorter CDS (Comeron et al. 1999; Betancourt and Presgraves 2002; Loewe and Charlesworth 2007), a trend opposite to that observed for testis-biased genes (Fig. 3A,B) (Hambuch and Parsch 2005). Thus, this suggests that the accumulation of non-optimal codons in testis-biased genes is influenced by factors other than genetic interference, and may reflect a different process such as selection. We therefore followed up this observation with detailed codon use analyses.

Moreover, it is notable that the extremely highly expressed testis-biased genes which were at least 5-fold sex-biased, tended to have a higher degree of sex-bias than the highly expressed testis-biased genes (the median was 149.5 and 210 for long and short genes in the former, and 71.0 and 69.5 in the latter, consistent with high extent of specialization in the testis in each group), suggesting the more-biased expression in the testis (among genes with ≥100 RPKM), the greater use of non-optimal codons

Non-optimal Codon use is Non-random in *D. melanogaster* Sex-biased Genes (Fig. S4)

We examined examples of non-optimal codons that had extremely high use, defined as RSCU ≥1.5, (that is, at least 1.5-fold higher than expected under equal codon use). For the Non-

$opt_{high\text{-}tRNAs}$ codons Thr-ACT and Gly-GGA, we found that 8.6% and 28.0% of the testis-biased genes had extreme use of these two respective codons, which was more than two-fold higher than that found for ovary-biased and unbiased genes (Chi-square tests P<0.05, Fig. S4A). For the Non-$opt_{low\text{-}tRNAs}$ Ile-ATA and Val-GTA, we found that more testis-biased genes had extreme use of these non-optimal codons than ovary-biased and unbiased genes. For example, nearly 4.0% of testis-biased genes had extremely high use of GTA, which was ten-fold more common than found in ovary-biased and unbiased genes (<0.4% of genes, MWU-tests P<0.05, Fig. S4A). Thus, genes with extreme use of non-optimal codons are increased in the testis-biased gene set.

It is worth noting that with respect to optimal codons, the percent of genes with extreme use of $Opt_{high\text{-}tRNAs}$ codons (RSCU ≥1.5) differed with respect to sex-biased gene sets, with examples shown in Fig. S4. Extreme use of the two $Opt_{high\text{-}tRNAs}$ codons Arg-GCG and Leu-CTG (shown in Fig. S4B) was found for 87.2% and 93.7% of the unbiased genes respectively, higher than the percentages found for ovary-biased and testis-biased genes (values between 56.1% and 89.1 %, Chi-square tests P< 0.05, Fig. S4B). A similar directional pattern was found for the Opt_{wobble} codons Pro-CCC and Ile-ATC, with extreme use being more common in unbiased and ovary-biased genes than in testis-biased genes (noting that the former codon had similar percentages for unbiased and ovary-biased genes. Fig. S4B, Chi-square for all other paired tests had P<0.05).

Notably, the two optimal wobble codons had markedly fewer genes with extreme use in the unbiased gene set, than the optimal codons with high-tRNAs. For example, the Opt_{wobble} codons CCC-Pro and Ile-ATC had extreme use in only 57.8% and 73.0% of unbiased genes (as compared to the 87.2% and 93.7% for $Opt_{high\text{-}tRNAs}$ Arg-GCG and Leu-CTG, Chi-square tests P<0.05), and a similar reduction in use of wobble optimal codons was found for ovary-biased and unbiased genes (Chi-square tests P<0.05, Fig. S4B). Thus, optimal wobble codons are less likely to exhibit extreme use in highly expressed sex-biased genes than optimal codons with plentiful tRNAs. Such a pattern may be a result of the fact that wobble codons have lower binding affinity to the tRNAs (Letzring et al. 2010; Stadler and Fire 2011; Quax et al. 2015; Stein and Frydman 2019), making them less efficient for translation than codons with plentiful tRNAs. In addition, it is also feasible that our findings for wobble optimal codons may indicate these codons are only used as warranted within an mRNA when required for the pacing of translation, and thus not as often used as optimal codons with plentiful tRNAs, which may ensure rapid and efficient translation (Akashi 2001; Quax et al. 2015). Overall, the patterns found here in Fig. S4B demonstrate that optimal wobble codons are not equivalent to optimal codons with plentiful tRNAs, and that the inclusion of tRNA analysis is warranted for future codon use studies, including in *D. melanogaster*, to allow these distinctions in optimal codon use.

Amino Acids Using Non-Optimal Codons Have High Surface Exposure and Disorder: Excluding and including Trp and Met (Fig. 2)

We compared Percent-Non-opt calculations to average RSA per gene across all amino acids. Given that Met and Typ are each encoded by one codon and were included in gene-wide RSA values, but not Percent-Non-opt calculations (which included all 18 amino acids with two or

more codons), we repeated the Percent-Non-opt calculations analysis with Met and Trp included and being scored as having optimal codons given their abundant tRNAs in Table S1. Using this approach, Percent-Non-opt and Percent-Opt across genes were each nearly perfectly correlated with the values obtained when excluding these two amino acids (Spearman's $R>0.99$, $P<10^{-7}$ for all gene sets). Thus, inclusion or exclusion of Met and Trp lead to similar results (Fig. 2A-F).

Individual Codons per Amino Acid and Direct Measurement of tRNA Abundances (referred to in Conclusions section)

Fig. S2 suggests co-evolution of amino acid use and tRNA gene numbers (pooled across all tRNAs) in the testis-biased, ovary-biased and unbiased genes. Thus, the patterns also suggest that the testes and ovaries may have similar tRNA populations at least at the whole amino acid level. However, individual tRNAs per amino acid could in theory still vary between the gonads. Direct quantification of synonymous tRNAs per amino acid in tissues/cell types in multicellular systems remains a challenge in terms of methodology and accuracy (due to higher structure of tRNAs, high similarity in tRNA sequences; and methods used including tRNA-seq, RNA-seq, RNA-polymerase occupancy on tRNA genes and microarrays (Dittmar et al. 2006; Guimaraes et al. 2020)). However, while certain available data from mammals and *Drosophila* suggest that tRNA abundances may vary among tissues (Moriyama and Powell 1997; Plotkin et al. 2004; Dittmar et al. 2006), other data suggest that tRNA populations may be invariant among some tissues types or cells (Dittmar et al. 2006; Schmitt et al. 2014; Rudolph et al. 2016; Stein and Frydman 2019). For example, recent analysis of genes in rapidly dividing and quiescent mouse fibroblast cells showed no differences in relative abundances of individual tRNAs (using tRNA-seq), despite marked differences in the gene codon use among cell types (Guimaraes et al. 2020). In addition, prior analysis of a subset of tRNAs using microarrays (the subset of tRNAs that were assessable using a microarray approach) in testes and ovaries in humans, showed the tRNA levels were similar between the two sex organs for at least some groups of amino acids (Dittmar et al. 2006). Thus, further data will help ascertain whether tRNA populations of synonymous codons within amino acids may vary between the gonads of *D. melanogaster*.

Supplementary Materials and Methods

Here, we provide additional details on the Materials and Methods from the main text.

Genome Datasets and Gonadal Expression Analyses

For our study, we downloaded the genome of *D. melanogaster* (13,986 protein coding genes, in version 6.50 (Gramates et al. 2022)). We extracted the longest CDS per gene for study. Before the assessment of sex-biased genes, we first identified and characterized the organismal optimal and non-optimal codons in *D. melanogaster*. Sets of optimal codons, those most used in highly expressed genes have been inferred in a number of studies in *D. melanogaster,* and have been uncorrelated to intron content (Shields et al. 1988), findings consistent with selection favoring optimal codon use (Shields et al. 1988; Moriyama and Powell 1997; Powell and Moriyama 1997; Duret and Mouchiroud 1999; Heger and Ponting 2007; Payne and Alvarez-Ponce 2019) (studies typically finding GC3-ending optimal codons; sometimes multiple codons were defined as optimal per amino acid, and usually excluding non-optimal codon and tRNA analysis). To define the optimal codons for our objectives herein, we compared the RSCU of highly expressed ribosomal protein genes (N=169) to that of the entire genome (downloaded RSCU from the CSD (Subramanian et al. 2022)), denoted as $\Delta RSCU_{Ribosome\text{-}All}$. The RSCU measures the relative frequency of a codon per synonymous codon family (amino acid) as compared to that expected under random use (e.g., expected value=1 under equal use, and higher and lower values indicate greater and lower use than expected (Sharp and Li 1987)). We defined the optimal codon per amino acid as the codon with the largest positive $\Delta RSCU_{Ribosome\text{-}All}$ (shown in Table S1, each RSCU from the CSD (Subramanian et al. 2022)), which in all cases was also classed as an optimal codon (called preferred therein) at the CSD with $P<0.05$ (which used genes with high and low effective number of codons (ENC) (Subramanian et al. 2022), see Notes in Table S1). The primary optimal codon for *D. melanogaster* using this approach was largely concordant with optimal codon lists obtained using ΔRSCU of the most highly versus the most lowly expressed genes defined previously (Duret and Mouchiroud 1999). This is another stringent method to identify optimal codons (Duret and Mouchiroud 1999; Sharp et al. 2005; Cutter et al. 2006; Ingvarsson 2008; Wang et al. 2011; Whittle et al. 2011; Whittle and Extavour 2015; Whittle et al. 2019; Whittle et al. 2021), which yielded optimal codons ending with G3 and C3 (and sometimes more than one optimal codon per amino acid (Shields et al. 1988; Moriyama and Powell 1997; Powell and Moriyama 1997; Heger and Ponting 2007; Payne and Alvarez-Ponce 2019)). However, we specifically identified a single optimal codon per amino acid herein, even for amino acids encoded by three or more synonymous codons, as typically one codon was much favored over all others (see the bolded ribosomal gene RSCU values versus un-bolded per amino acid and $\Delta RSCU_{Ribosome\text{-}All}$, Table S1). In sum, a G3 or C3 optimal codon, one per amino acid, was identified from our criteria (Table S1) that had elevated use in highly expressed genes relative to the least expressed genes in the genome (Duret and Mouchiroud 1999; Subramanian et al. 2022), and relative to whole genome (Table S1).

Non-optimal codons, whose identities have been much less studied in *D. melanogaster* to date, were defined herein as all those codons per amino acid that were not the optimal codon. This

classification approach was supported by the facts that first, we found that all of the non-optimal codons typically had a negative value or near-zero value for $\Delta RSCU_{Ribosome\text{-}All}$ (Table S1), and second, each non-optimal codon had a much lower absolute RSCU value in the ribosomal protein genes than the optimal codon (typically at least 1.5 fold smaller than the optimal codon, Table S1), and in some cases had a value in excess of six fold smaller than the optimal codon, thus confirming much lower use in highly expressed versus genome-wide genes (for example, for Val, RSCU for the optimal codon GTG was 1.84, but was only 0.27 for its sister non-optimal codon GTA). A total of 18 optimal and 41 non-optimal codons across 18 amino acids were identified and studied (Table S1). Further, we then classified the optimal codons and the non-optimal codons each into two groups, based on the presence of few or of plentiful exact matching tRNA gene copies in the genome, and studied their use with respect to sex-biased expression.

tRNA Genes in the Genome per Codon

The tRNA gene counts in an organism's genome have provided an effective proxy for the typical relative cellular tRNA abundances (Ikemura 1981; Ikemura 1985; Sharp et al. 1986; Percudani et al. 1997; Duret 2000; Akashi 2001; Cognat et al. 2008; Du et al. 2017; Whittle et al. 2019; Whittle et al. 2021). Thus, we obtained the number of genomic tRNAs per codon type for *D. melanogaster* from the GtRNA database ((http://gtrnadb.ucsc.edu) (Chan and Lowe 2016), which utilizes tRNAscan-SE to extract tRNA genes from the genome (Lowe and Chan 2016; Chan and Lowe 2019)).

Identification of sex-biased genes

To identify sex-biased genes, we used the modEncode database, which contains expression data (Graveley et al. 2011; Gramates et al. 2022) for a wide range of *D. melanogaster* tissue types and developmental stages (N=59 tissues/stages herein, Table S2 (Graveley et al. 2011; Li et al. 2014; Gramates et al. 2022). The entire expression dataset (Table S2) was used to measure *tau* across tissues (see below, (Yanai et al. 2005)), and thus all expression data were obtained from a single source. For the identification of sex-biased genes, we compared expression of 4-day mated testes and 4-day mated ovaries. First, from examining testes and ovaries, we identified all genes with five-fold or higher expression bias in the gonad of one sex than in the other, which is a high threshold for defining sex-bias (Proschel et al. 2006; Whittle et al. 2007; Mank et al. 2008; Meisel 2011; Assis et al. 2012). Further, for the sex-biased gene set, we only included genes that had RPKM≥100 (in the testes or the ovaries) to ensure there was high expression in at least one sex, such that a high sex-bias ratio was not obtained from two weak expression values. The cutoff of 100 RPKM was at the 90th and 95th percentiles of RPKM for genes from the testes and ovaries respectively (mean expression (RPKM) for the gene set per tissue type was 389.8 ± 24.3 and 349.5 ± 36.4, t-test p=0.42), affirming that highly expressed sex-biased genes were under study. Testis- and ovary-biased genes included genes that were sex-specific, or without detectable expression in the other tissue (Fig. S1). Unbiased genes were identified as those that had ≥100 RPKM in either the testes or ovaries and no sex-bias (<5-fold difference, and thus given the cutoff of 100 RPKM

in one sexual tissue, had at least 20 RPKM in the opposite tissue type), thus ensuring expression in both sexes. GO analysis was conducted for testis- and ovary-biased genes in DAVID (Sherman et al. 2022) to verify the identified gene sets were associated with testis and ovaries.

Male and female reproductive expression datasets were also available from modEncode (Graveley et al. 2011; Li et al. 2014) for male accessory glands, which produce the seminal fluid proteins (Sepil et al. 2019), and for the virgin female ovaries (Table S2). Thus, for additional rigor, we assessed the fraction of the identified sex-biased genes in our main analysis of gonads of mated tissues were at least five-fold male-biased when using contrasts of the accessory glands vs virgin ovary tissues.

Comparison of Optimal and Non-optimal Codon Use in Sex-biased Genes

The finest scale to study codon use is on an individual codon-by-codon basis; for example, sex-differences in codon use for each of 18 optimal codons and each of the 41 non-optimal codons. For our analysis of sex-biased genes, we determined the RSCU for every codon for each gene in the testis-biased, ovary-biased and unbiased genes using the program CodonW (Peden 1999). Then, for each of the 59 codons under study, we determined $\Delta RSCU_{Testis\text{-}Ovary}$ and as $\Delta RSCU_{Testis\text{-}Unbiased}$ across all genes per gene set, as described in the main text (Whittle et al. 2007).

Comparisons Among Sex-biased Gene Sets using Codon Use Gene Indices

To initially screen the level of optimal codon and non-optimal codon use in the sex-biased gene sets we used the codon adaptation index (CAI) and the frequency of optimal codons (Fop) (Bahiri-Elitzur and Tuller 2021), which broadly measure codon adaptation for optimal codons, and which do not include tRNA analysis. CAI provides a score of codon use for each gene (0 to 1) based on codon frequencies in the highly expressed reference gene set (Subramanian et al. 2022), and is thought to reflect adaptation in codon use to gene expression (Sharp and Li 1987; Bahiri-Elitzur and Tuller 2021). Fop comprises an index of how often optimal codons are used in each gene under study (0 for no optimal codons used for a gene, 1 for only optimal codons) (Ikemura 1981). We found CAI and Fop values were strongly correlated across the *D. melanogaster* genome (Spearman's R=0.967, $P<10^{-7}$), and thus solely used CAI values for analysis, which were obtained from the CSD (Subramanian et al. 2022).

CAI and Fop have typically been used to assess codon adaptation in terms of optimal codon use in genes (Ikemura 1981; Sharp and Li 1987; Bahiri-Elitzur and Tuller 2021), including in *D. melanogaster* (Hambuch and Parsch 2005), and thus are indicators of improved translational efficiency (with values closer to 1). However, lower values in codon bias indices innately indicate increased non-optimal codon use, which accompanies a reduction of optimal codons (Zhou et al. 2015). Thus, herein lower values of CAI were taken as evidence of enhanced use of non-optimal codons per gene.

Protein Folding, Relative Solvent Accessibility and Codon Use in the Sex-biased Genes

The RSA value reflects the degree of burial or solvent exposure of an amino acid based on its local secondary and tertiary structures, or lack of well-folded structures, relative to its maximum

possible surface area per amino acid (in Angstroms) (Braun 1998; Tien et al. 2013). To determine the RSA per amino acid we used NetsurfP v3.0 at default parameters (Hoie et al. 2022). In addition to RSA, we also determined the type of secondary structure that each amino acid was a part of, that is, an alpha-helix, string (β-string/sheet) or a random coil (typically disordered) using NetsurfP 3.0 (Hoie et al. 2022).

Protein folding predictions for *D. melanogaster* proteins (Uniprot proteome Identity UP000000803, taxon identity = 7227) were determined using Alphafold2 (Juniper et al. 2021), and are available from the AlphaFold2 database for most proteins (https://alphafold.ebi.ac.uk)(Varadi et al. 2022). We recognize that the accuracy of predicted formations for disordered regions may sometimes be less well statistically supported than for well-structured regions in the database (Baek and Kepp 2022; Varadi et al. 2022).

Expression Specificity, or *tau*

While gene expression level has been associated with the use of optimal codons, and with translational efficiency of abundant transcripts (Ikemura 1985; Shields et al. 1988; Akashi 2001; Hambuch and Parsch 2005; Behura and Severson 2011; Wang et al. 2011; Galtier et al. 2018; Whittle et al. 2019; Whittle et al. 2021), a factor worth consideration herein with a possible effect on codon use is expression breadth. Thus, we used data on the expression specificity index *tau* for each studied gene in the *D. melanogaster* genome using gene expression data available across the 59 tissues/stages from the modEncode database (Table S2 (Graveley et al. 2011; Gramates et al. 2022) as per (Yanai et al. 2005) (see also our prior assessment of *tau* in (Whittle and Extavour 2023)). *tau* values can range from 0 to 1, where values nearer to 1 indicate high expression specificity and lower values denote greater expression breadth across a range of tissue types (Yanai et al. 2005; Whittle and Extavour 2023). As shown in Table S2, the data included gene expression levels (RPKM) across development for the embryos (12 stages), larvae (6 stages), pupae (6 stages) and adults (3 stages of males/females), and for tissue types of the adult males and females. For each gene, the *tau* value was measured as follows:

$$\boldsymbol{tau\ (\tau) = \sum_{i=1}^{n}(1-\hat{x}_i)/(n-1);\ \hat{x}_i = x_i/max\ (x_i)}$$

where n=number of tissues/stages, i=tissue/stage, x_i= transcription level of gene in tissue/stage i, and max (x_i)= the transcription level in the tissue/stage type with maximum expression (Yanai et al. 2005).

Supplementary References

Akashi H. 2001. Gene expression and molecular evolution. Curr Opin Genet Dev. 11:660–666

Argyridou E, Parsch J. 2018. Regulation of the X Chromosome in the Germline and Soma of,Drosophila melanogaster,Males. Genes. 9(5):242 (doi.org/10.3390/genes9050242) https://www.ncbi.nlm.nih.gov/pubmed/29734690. https://doi.org/10.3390/genes9050242

Assis R, Zhou Q, Bachtrog D. 2012. Sex-biased transcriptome evolution in Drosophila. Genome Biol Evol. 4(11):1189–200 http://www.ncbi.nlm.nih.gov/pubmed/23097318. https://doi.org/10.1093/gbe/evs093

Baek KT, Kepp KP. 2022. Assessment of AlphaFold2 for Human Proteins via Residue Solvent Exposure. J Chem Inf Model. 62(14):3391–3400 https://www.ncbi.nlm.nih.gov/pubmed/35785970. https://doi.org/10.1021/acs.jcim.2c00243

Bahiri-Elitzur S, Tuller T. 2021. Codon-based indices for modeling gene expression and transcript evolution. Comput Struct Biotechnol J. 19:2646–2663 https://www.ncbi.nlm.nih.gov/pubmed/34025951. https://doi.org/10.1016/j.csbj.2021.04.042

Behura SK, Severson DW. 2011. Coadaptation of isoacceptor tRNA genes and codon usage bias for translation efficiency in,Aedes aegypti,and,Anopheles gambiae. Insect Mol Biol. 20:177–187

Betancourt AJ, Presgraves DC. 2002. Linkage limits the power of natural selection in Drosophila. Proc Natl Acad Sci U S A. 99(21):13616–13620. https://doi.org/10.1073/pnas.212277199

Braun WF R,. 1998. Exact and efficient analytical calculation of the accessible surface areas and their gradients for macromolecules. J Comput Chem. 19:319–333

Chan PP, Lowe TM. 2016. GtRNAdb 2.0: an expanded database of transfer RNA genes identified in complete and draft genomes. Nucleic Acids Res. 44(D1):D184-9 https://www.ncbi.nlm.nih.gov/pubmed/26673694. https://doi.org/10.1093/nar/gkv1309

Chan PP, Lowe TM. 2019. tRNAscan-SE: Searching for tRNA Genes in Genomic Sequences. Methods Mol Biol. 1962:1–14 https://www.ncbi.nlm.nih.gov/pubmed/31020551. https://doi.org/10.1007/978-1-4939-9173-0_1

Cognat V et al. 2008. On the evolution and expression of,Chlamydomonas reinhardtii,nucleus-encoded transfer RNA genes. Genetics. 179(1):113–23 https://www.ncbi.nlm.nih.gov/pubmed/18493044. https://doi.org/10.1534/genetics.107.085688

Comeron JM, Kreitman M, Aguade M. 1999. Natural selection on synonymous sites is correlated with gene length and recombination in,Drosophila. Genetics. 151:239–49 http://www.ncbi.nlm.nih.gov/pubmed/9872963

Cutter AD, Wasmuth JD, Blaxter ML. 2006. The evolution of biased codon and amino acid usage in nematode genomes. Mol Biol Evol. 23(12):2303–15 http://www.ncbi.nlm.nih.gov/pubmed/16936139. https://doi.org/10.1093/molbev/msl097

Dittmar KA, Goodenbour JM, Pan T. 2006. Tissue-specific differences in human transfer RNA expression. PLoS Genet. 2(12):e221 https://www.ncbi.nlm.nih.gov/pubmed/17194224. https://doi.org/10.1371/journal.pgen.0020221

Du MZ et al. 2017. Co-adaption of tRNA gene copy number and amino acid usage influences translation rates in three life domains. DNA Res. 24(6):623–633 https://www.ncbi.nlm.nih.gov/pubmed/28992099. https://doi.org/10.1093/dnares/dsx030

Dufton MJ. 1997. Genetic code synonym quotas and amino acid complexity: cutting the cost of proteins? J Theor Biol. 187(2):165–73 https://www.ncbi.nlm.nih.gov/pubmed/9237887. https://doi.org/10.1006/jtbi.1997.0443

Duret L. 2000. tRNA gene number and codon usage in the,C. elegans,genome are co-adapted for optimal translation of highly expressed genes. Trends Genet. 16:287–9 http://www.ncbi.nlm.nih.gov/pubmed/10858656

Duret L, Mouchiroud D. 1999. Expression pattern and, surprisingly, gene length shape codon usage in Caenorhabditis, Drosophila, and Arabidopsis. Proc Natl Acad Sci. 96(8):4482–4487 http://www.ncbi.nlm.nih.gov/pubmed/10200288. https://doi.org/10.1073/pnas.96.8.4482

Fleming J et al. 2025. AlphaFold Protein Structure Database and 3D-Beacons: New Data and Capabilities. J Mol Biol. 437(15):168967. https://doi.org/10.1016/j.jmb.2025.168967

Galtier N et al. 2018. Codon Usage Bias in Animals: Disentangling the Effects of Natural Selection, Effective Population Size, and GC-Biased Gene Conversion. Mol Biol Evol. 35(5):1092–1103 https://www.ncbi.nlm.nih.gov/pubmed/29390090. https://doi.org/10.1093/molbev/msy015

Gramates LS et al. 2022. FlyBase: a guided tour of highlighted features. Genetics. 220(4):iyac035 https://www.ncbi.nlm.nih.gov/pubmed/35266522. https://doi.org/10.1093/genetics/iyac035

Graveley BR et al. 2011. The developmental transcriptome of Drosophila melanogaster. Nature. 471(7339):473–9 http://www.ncbi.nlm.nih.gov/entrez/query.fcgi?cmd=Retrieve&db=PubMed&dopt=Citation&list_uids=21179090. https://doi.org/10.1038/nature09715

Guimaraes JC et al. 2020. A rare codon-based translational program of cell proliferation. Genome Biol. 21(1):44 https://www.ncbi.nlm.nih.gov/pubmed/32102681. https://doi.org/10.1186/s13059-020-1943-5

Hambuch TM, Parsch J. 2005. Patterns of synonymous codon usage in Drosophila melanogaster genes with sex-biased expression. Genetics. 170(4):1691–700 https://www.ncbi.nlm.nih.gov/pubmed/15937136. https://doi.org/10.1534/genetics.104.038109

Heger A, Ponting CP. 2007. Variable strength of translational selection among 12 Drosophila species. Genetics. 177(3):1337–48 https://www.ncbi.nlm.nih.gov/pubmed/18039870. https://doi.org/10.1534/genetics.107.070466

Hill WG, Robertson A. 1966. The effect of linkage on limits to artificial selection. Genet Res. 8:269–94 https://www.ncbi.nlm.nih.gov/pubmed/5980116

Hoie MH et al. 2022. NetSurfP-3.0: accurate and fast prediction of protein structural features by protein language models and deep learning. Nucleic Acids Res. 50(W1):W510–W515 https://www.ncbi.nlm.nih.gov/pubmed/35648435. https://doi.org/10.1093/nar/gkac439

Ikemura T. 1981. Correlation between the abundance of Escherichia coli transfer RNAs and the occurrence of the respective codons in its protein genes: a proposal for a synonymous codon choice that is optimal for the E. coli translational system. J Mol Biol. 151(3):389–409 https://www.ncbi.nlm.nih.gov/pubmed/6175758. https://doi.org/10.1016/0022-2836(81)90003-6

Ikemura T. 1985. Codon usage and tRNA content in unicellular and multicellular organisms. Molecular Biology and Evolution. 2:13–34 http://www.ncbi.nlm.nih.gov/pubmed/3916708

Ingvarsson PK. 2007. Gene expression and protein length influence codon usage and rates of sequence evolution in Populus tremula. Mol Biol Evol. 24(3):836–44 https://www.ncbi.nlm.nih.gov/pubmed/17204548. https://doi.org/10.1093/molbev/msl212

Ingvarsson PK. 2008. Molecular evolution of synonymous codon usage in Populus. BMC Evol Biol. 8(1):307 http://www.ncbi.nlm.nih.gov/pubmed/18983655. https://doi.org/10.1186/1471-2148-8-307

Jumper J et al. 2021. Highly accurate protein structure prediction with AlphaFold. Nature. 596(7873):583–589. https://doi.org/10.1038/s41586-021-03819-2

Juniper T et al. 2021. Use of beta-D-glucan in diagnosis of suspected Pneumocystis jirovecii pneumonia in adults with HIV infection. Int J STD AIDS. 32(11):1074–1077 https://www.ncbi.nlm.nih.gov/pubmed/34106017. https://doi.org/10.1177/09564624211022247

Letzring DP, Dean KM, Grayhack EJ. 2010. Control of translation efficiency in yeast by codon-anticodon interactions. RNA. 16(12):2516–28 https://www.ncbi.nlm.nih.gov/pubmed/20971810. https://doi.org/10.1261/rna.2411710

Li JJ, Huang H, Bickel PJ, Brenner SE. 2014. Comparison of D. melanogaster and C. elegans developmental stages, tissues, and cells by modENCODE RNA-seq data. Genome Res. 24(7):1086–101 http://www.ncbi.nlm.nih.gov/pubmed/24985912. https://doi.org/10.1101/gr.170100.113

Loewe L, Charlesworth B. 2007. Background selection in single genes may explain patterns of codon bias. Genetics. 175(3):1381–93 https://www.ncbi.nlm.nih.gov/pubmed/17194784. https://doi.org/10.1534/genetics.106.065557

Lowe TM, Chan PP. 2016. tRNAscan-SE On-line: integrating search and context for analysis of transfer RNA genes. Nucleic Acids Res. 44(W1):W54-7 https://www.ncbi.nlm.nih.gov/pubmed/27174935. https://doi.org/10.1093/nar/gkw413

Mank JE, Hultin-Rosenberg L, Webster MT, Ellegren H. 2008. The unique genomic properties of sex-biased genes: insights from avian microarray data. BMC Genomics. 9(1):148 https://www.ncbi.nlm.nih.gov/pubmed/18377635. https://doi.org/10.1186/1471-2164-9-148

Meisel RP. 2011. Towards a more nuanced understanding of the relationship between sex-biased gene expression and rates of protein-coding sequence evolution. Mol Biol Evol. 28(6):1893–900 https://www.ncbi.nlm.nih.gov/pubmed/21239389. https://doi.org/10.1093/molbev/msr010

Moriyama EN, Powell JR. 1997. Codon usage bias and tRNA abundance in,Drosophila. J Mol Evol. 45:514–23 https://www.ncbi.nlm.nih.gov/pubmed/9342399

Parisi M et al. 2003. Paucity of genes on the,Drosophila,X chromosome showing male-biased expression. Science. 299(5607):697–700. https://doi.org/10.1126/science.1079190

Payne BL, Alvarez-Ponce D. 2019. Codon usage differences among genes expressed in different tissues of,Drosophila melanogaster. Genome Biol Evol. 11(4):1054–1065 https://www.ncbi.nlm.nih.gov/pubmed/30859203. https://doi.org/10.1093/gbe/evz051

Peden JF. 1999. Analysis of codon usage. University of Nottingham.

Percudani R, Pavesi A, Ottonello S. 1997. Transfer RNA gene redundancy and translational selection in Saccharomyces cerevisiae. J Mol Biol. 268(2):322–30 https://www.ncbi.nlm.nih.gov/pubmed/9159473. https://doi.org/10.1006/jmbi.1997.0942

Plotkin JB, Robins H, Levine AJ. 2004. Tissue-specific codon usage and the expression of human genes. Proc Natl Acad Sci U S A. 101(34):12588–91 https://www.ncbi.nlm.nih.gov/pubmed/15314228. https://doi.org/10.1073/pnas.0404957101

Powell JR, Moriyama EN. 1997. Evolution of codon usage bias in,Drosophila. Proc Natl Acad Sci U S A. 94(15):7784–90 https://www.ncbi.nlm.nih.gov/pubmed/9223264. https://doi.org/10.1073/pnas.94.15.7784

Proschel M, Zhang Z, Parsch J. 2006. Widespread adaptive evolution of Drosophila genes with sex-biased expression. Genetics. 174(2):893–900 https://www.ncbi.nlm.nih.gov/pubmed/16951084. https://doi.org/10.1534/genetics.106.058008

Quax T, Claassens N, D Soll, Ooost J van der. 2015. Codon Bias as a Means to Fine-Tune Gene Expression. Mol Cell. 59:149–161

Rudolph KL et al. 2016. Codon-Driven Translational Efficiency Is Stable across Diverse Mammalian Cell States. PLoS Genet. 12(5):e1006024 https://www.ncbi.nlm.nih.gov/pubmed/27166679. https://doi.org/10.1371/journal.pgen.1006024

Schmitt BM et al. 2014. High-resolution mapping of transcriptional dynamics across tissue development reveals a stable mRNA-tRNA interface. Genome Res. 24(11):1797–807 https://www.ncbi.nlm.nih.gov/pubmed/25122613. https://doi.org/10.1101/gr.176784.114

Sepil I et al. 2019. Quantitative Proteomics Identification of Seminal Fluid Proteins in Male,Drosophila melanogaster. Mol Cell Proteom. 18(Suppl 1):S46–S58 https://www.ncbi.nlm.nih.gov/pubmed/30287546. https://doi.org/10.1074/mcp.ra118.000831

Sharp PM et al. 2005. Variation in the strength of selected codon usage bias among bacteria. Nucleic Acids Res. 33(4):1141–53 https://www.ncbi.nlm.nih.gov/pubmed/15728743. https://doi.org/10.1093/nar/gki242

Sharp PM, Li WH. 1987. The codon Adaptation Index--a measure of directional synonymous codon usage bias, and its potential applications. Nucleic Acids Res. 15(3):1281–95 https://www.ncbi.nlm.nih.gov/pubmed/3547335. https://doi.org/10.1093/nar/15.3.1281

Sharp PM, Tuohy TM, Mosurski KR. 1986. Codon usage in yeast: cluster analysis clearly differentiates highly and lowly expressed genes. Nucleic Acids Res. 14:5125–43 http://www.ncbi.nlm.nih.gov/pubmed/3526280

Sherman BT et al. 2022. DAVID: a web server for functional enrichment analysis and functional annotation of gene lists (2021 update). Nucleic Acids Res. 50(W1):W216–W221 https://www.ncbi.nlm.nih.gov/pubmed/35325185. https://doi.org/10.1093/nar/gkac194

Shields DC, Sharp PM, Higgins DG, Wright F. 1988. “Silent” sites in Drosophila genes are not neutral: evidence of selection among synonymous codons. Molecular biology and evolution. 5:704–16 http://www.ncbi.nlm.nih.gov/pubmed/3146682

Stadler M, Fire A. 2011. Wobble base-pairing slows in vivo translation elongation in metazoans. RNA. 17(12):2063–73 https://www.ncbi.nlm.nih.gov/pubmed/22045228. https://doi.org/10.1261/rna.02890211

Stein KC, Frydman J. 2019. The stop-and-go traffic regulating protein biogenesis: How translation kinetics controls proteostasis. J Biol Chem. 294(6):2076–2084 https://www.ncbi.nlm.nih.gov/pubmed/30504455. https://doi.org/10.1074/jbc.rev118.002814

Subramanian K, Payne B, Feyertag F, Alvarez-Ponce D. 2022. The Codon Statistics Database: A Database of Codon Usage Bias. Mol Biol Evol. 39(8):msac157 https://www.ncbi.nlm.nih.gov/pubmed/35859338. https://doi.org/10.1093/molbev/msac157

Tien MZ et al. 2013. Maximum allowed solvent accessibilities of residues in proteins. PLoS One. 8(11):e80635 https://www.ncbi.nlm.nih.gov/pubmed/24278298. https://doi.org/10.1371/journal.pone.0080635

Varadi M et al. 2022. AlphaFold Protein Structure Database: massively expanding the structural coverage of protein-sequence space with high-accuracy models. Nucleic Acids Res. 50(D1):D439–D444 https://www.ncbi.nlm.nih.gov/pubmed/34791371. https://doi.org/10.1093/nar/gkab1061

Wang B et al. 2011. Optimal codon identities in bacteria: implications from the conflicting results of two different methods. PLoS One. 6:e22714

Whittle CA, Extavour CG. 2015. Codon and amino acid usage are shaped by selection across divergent model organisms of the Pancrustacea. G3: Genes, Genomes, Genet. 5(11):2307–2321. https://doi.org/10.1534/g3.115.021402

Whittle CA, Extavour CG. 2023. Revealing the links between gene protein sequence evolution and the rapid divergence of ovariole numbers in,Drosophila. Biorxiv. [published online ahead of print]

Whittle CA, Kulkarni A, Chung N, Extavour CG. 2021. Adaptation of codon and amino acid use for translational functions in highly expressed cricket genes. BMC Genomics. 22(1):234 https://www.ncbi.nlm.nih.gov/pubmed/33823803. https://doi.org/10.1186/s12864-021-07411-w

Whittle CA, Kulkarni A, Extavour CG. 2019. Evidence of multifaceted functions of codon usage in translation within the model beetle Tribolium castaneum. DNA Res. 26(6):473–484 https://www.ncbi.nlm.nih.gov/pubmed/31922535. https://doi.org/10.1093/dnares/dsz025

Whittle CA, Kulkarni A, Extavour CG. 2020. Absence of a faster-X effect in beetles (,Tribolium,, Coleoptera). G3 (Bethesda). 10:1125–1136

Whittle CA, Malik MR, Krochko JE. 2007. Gender-specific selection on codon usage in plant genomes. BMC Genomics. 8:169–179

Whittle CA, Sun Y, Johannesson H. 2011. Evolution of synonymous codon usage in,Neurospora tetrasperma,and,Neurospora discreta. Genome Biol Evol. 3:332–343

Yanai I et al. 2005. Genome-wide midrange transcription profiles reveal expression level relationships in human tissue specification. Bioinformatics. 21(5):650–9 https://www.ncbi.nlm.nih.gov/pubmed/15388519. https://doi.org/10.1093/bioinformatics/bti042

Zhang Z, Hambuch TM, Parsch J. 2004. Molecular evolution of sex-biased genes in,Drosophila. Mol Biol Evol. 21(11):2130–9 https://www.ncbi.nlm.nih.gov/pubmed/15282334. https://doi.org/10.1093/molbev/msh223

Zhou M et al. 2015. Nonoptimal codon usage influences protein structure in intrinsically disordered regions. Mol Microbiol. 97(5):974–87 https://www.ncbi.nlm.nih.gov/pubmed/26032251. https://doi.org/10.1111/mmi.13079
